# Evaluating Leadership Dynamics: A Comprehensive Cross-Sectional Study of Madinah's Primary Healthcare Centers

**DOI:** 10.7759/cureus.67199

**Published:** 2024-08-19

**Authors:** Muhannad A Abuljood, Mohammed Almatrafi, Ali Alsolami, Yousef Aloufi, Ahmed Abualkhair

**Affiliations:** 1 Preventive Medicine Program for Postgraduate Studies, Ministry of Health, Madinah, SAU; 2 Preventive Medicine, General Directorate of Health Affairs of Medina, Madinah, SAU; 3 Public Health Administration, Ministry of Health, Jeddah, SAU; 4 Preventive Medicine, Al Madinah Health Cluster, Ministry of Health, Madinah, SAU; 5 Public Health, King Salman Medical City, Madinah, SAU

**Keywords:** healthcare management, performance outcome, leadership effectiveness, health care leadership, healthcare reform, staff satisfaction, leadership in healthcare management, kingdom of saudi arabia (ksa), leadership styles, primary healthcare

## Abstract

Objectives: This study focuses on the effectiveness of healthcare leadership in primary healthcare centers in Madinah, Saudi Arabia, examining the interplay between leadership styles, emotional intelligence, and their impact on leadership effectiveness. Emphasizing the critical role of primary healthcare as outlined by the World Health Organization, the research addresses the gap in understanding leadership dynamics within the Saudi healthcare context.

Methods: A cross-sectional design was used, involving 89 managers and deputies from 48 primary healthcare centers in Madinah City, Saudi Arabia in 2023. The methodology included a comprehensive questionnaire assessing leadership effectiveness, emotional intelligence, and socio-demographic variables. Data analysis was performed using SPSS version 26 (Armonk, NY: IBM Corp), incorporating chi-square tests, t-tests, analysis of variance (ANOVA), Pearson correlation, and binary logistic regression for statistical evaluation.

Results: The findings revealed a high level of leadership effectiveness (79.8%; 95% CI: 62.7±10.2) among the study participants. Emotional intelligence emerged as a significant factor in effective leadership, evidenced by a strong positive correlation (r=0.75; p=0.001) between emotional intelligence and leadership effectiveness. The study also observed a predominant preference for democratic leadership styles among participants, with no significant variance in leadership effectiveness across different styles. A notable area for improvement identified was self-awareness among healthcare leaders.

Conclusion: The research concludes that effective healthcare leadership, significantly influenced by emotional intelligence, is essential for reaching high-quality primary healthcare. It advocates for the integration of emotional intelligence training, especially focusing on self-awareness, in leadership development programs for healthcare professionals.

## Introduction

Primary health care (PHC), as defined by the World Health Organization (WHO) [1], constitutes essential healthcare accessible to all people based on scientifically sound and socially acceptable methods. In Saudi Arabia, the Ministry of Health (MOH) [2] oversees a comprehensive healthcare system, including primary, secondary, and tertiary care, focusing on preventive, curative, and rehabilitative needs [3]. With more than 2300 PHC centers, PHC serves as the backbone of the Kingdom's healthcare plan, undergoing substantial evolution to enhance its efficacy and reach [2,4]. Within this context, leadership in health care emerges as a critical and dynamic component that directly influences individual interactions and the achievement of collective goals across various fields. Effective leadership, characterized by robust communication and morale-boosting capabilities, significantly impacts healthcare delivery and outcomes [5-7].

Recent studies have shed light on the impact of leadership styles on healthcare center performance, staff satisfaction, and overall healthcare reform. For instance, a study in Riyadh evaluated leadership styles in PHC centers using a multifactor leadership questionnaire, revealing a correlation between transformational leadership and performance outcomes [8]. Similarly, research in China on vertical integration in health care has emphasized the role of leadership in enhancing professional competency and patient satisfaction [9]. In Al-Jouf, Saudi Arabia, a study demonstrated a strong link between leadership styles and staff job satisfaction in PHC centers, underscoring the importance of effective leadership in healthcare settings [10]. Moreover, a review of Saudi Arabia's PHC reform highlighted the positive progression in healthcare services, attributing this improvement to leadership and management initiatives [11]. This sentiment is echoed in another study comparing districts with and without leadership, management, and governance (LMG), where districts with LMG showcased superior performance [12]. Additionally, a study on the self-perception of leadership styles among PHC managers emphasized the varying impacts of different leadership approaches on job satisfaction and effectiveness [5].

These studies collectively emphasize the pivotal role of leadership in shaping the healthcare landscape but none mentions what factors attribute to effective leadership. The primary aim of this research is to delve into various PHC managerial approaches in Saudi Arabia, evaluate their effectiveness, and identify the optimal leadership style for PHC managers. This endeavor aligns with Saudi Arabia's Vision 2030, which aims to enhance healthcare leadership competencies and, consequently, improve healthcare system outcomes [13].

This article was previously posted to the medRxiv preprint server on April 12th, 2024.

## Materials and methods

This cross-sectional study was conducted among managers and their deputies in 48 PHC centers in Madinah City, Saudi Arabia in 2023-2024 to determine the effectiveness of healthcare leadership. Using OpenEpi Version 3, the sample size was estimated to be 85 participants, based on a 50% prevalence of leadership effectiveness among the 48 PHC centers in Madinah, a 5% margin of error, with a 95% confidence level and a 10% nonresponse rate. Participants were selected through a questionnaire distributed electronically that targeted both male and female managers and deputies in Madinah's PHC centers. The exclusion criteria included managers or deputies outside of Madinah or those on annual leave during the study period. The data were collected from November 2023 to April 2024. The healthcare leadership effectiveness of managers was measured using a questionnaire adapted from the Management Research Group (MRG). Calculated Cronbach alpha (α) to check the reliability of effective leadership measuring tool got a value of 0.78 [14]. Quick Emotional Intelligence Self-Assessment (QEISA) tool adapted from the 2015 Paul Mohapel model was used and checked the reliability of the emotional intelligence assessment tool through calculating Cronbach alpha (α) and we found the Cronbach alpha (α) value of 0.81 [14]. The outcome variable, leadership effectiveness, was measured alongside sociodemographic characteristics using a 15-item questionnaire covering three dimensions of leadership: vision creation, implementation of vision, and developing followership [15]. Emotional intelligence was assessed through self-awareness, emotional management, social awareness, and relationship management using a 40-item self-report questionnaire [16]. For each item, a five-point Likert scale was used, and emotional intelligence was categorized as low, moderate, or high based on predefined criteria [17]. The data were analyzed using SPSS version 26 (Armonk, NY: IBM Corp), employing descriptive statistics, chi-square tests, t-tests, analysis of variance (ANOVA), and Pearson correlation for quantitative and qualitative variables. A binary logistic regression model was used to identify factors associated with effective leadership. Ethical approval was obtained from the Al Madinah Health Cluster, and informed consent was secured from all participants, ensuring the anonymity of the collected data.

## Results

A total of 89 PHC managers and their deputies participated in this cross-sectional study. The demographic profile revealed that a majority of the participants were male, 84 (94.4%), with a predominant age group of 35-45 years, 65 (74.1%). Regarding family size, approximately half of the participants, 45 (50.6%), had between five and seven household members. Among the respondents, 52 (58.4%) were PHC managers, 37 (41.6%) were deputies, more than half, 50 (56.2%), held diplomas, and 34 (28.1%) had a bachelor’s degree or higher. Work experience of more than 15 years was common among 55 (61.8%) participants, and most, 65 (73%), earned salaries between 10,000 and 20,000 SAR. A significant proportion of participants, 79 (88%), preferred democratic leadership styles, 8 (9%) leaned toward laissez-faire styles, and a minority, 2 (2.2%), adopted autocratic styles; nearly two-thirds had attended leadership and management courses.

In terms of emotional intelligence, Table 1 shows more than two-thirds, 70 (78.7%), of the participants exhibited a high level, 19 (21.3%) had a moderate level, and none had a low level. The analysis of emotional intelligence dimensions revealed that self-awareness (mean 20.9±6.8) was relatively weaker than relationship management (mean 33.1±6.7). Regarding leadership effectiveness, Table 2 shows that 71 (79.8%) of the PHC managers and deputies demonstrated high effectiveness, while 18 (20.2%) showed low effectiveness. The dimension of implementing organizational vision scored the highest in effectiveness (mean 21.7±4.7). No significant differences were observed between the low- and high-healthcare-effectiveness groups in terms of sociodemographic characteristics such as age, sex, marital status, family size, or education. However, Table 3 shows that a notable proportion of high-efficacy group participants, 44 (62%), had more than 15 years of work experience. Leadership styles and attendance in leadership courses were more common in the high-efficacy group, yet the difference was not significant. Participants with high emotional intelligence were significantly more prevalent in the high-leadership-effectiveness group (91.5%, p=0.001).

**Table 1:** Frequency distribution of emotional intelligence dimensions among managers and their deputies in primary healthcare centers in Madinah/KSA (n=89) Total score in each domain is classified according to the following criteria: 0-24: low; 25-34: moderate; 35-44: high. The total emotional Intelligence score is classified as follows: low emotional intelligence (<53), moderate (53- 106), and high emotional intelligence >106 [14].

Variables	Frequency	Percent	Min-Max	Mean±SD
Self-awareness				
Low	62	69.7	3-36	20.9±6.8
Moderate	25	28.1		
High	2	2.2		
Emotional management				
Low	9	10.1	10-40	32.3±6.2
Moderate	44	49.4		
High	36	40.4		
Social awareness				
Low	11	12.4	17-40	31.7±5.9
Moderate	43	48.3		
High	35	39.3		
Relationship management				
Low	10	11.2	9-40	33.1±6.7
Moderate	32	36.0		
High	47	52.8		
Total emotional Intelligence				
Low	0	0	58-154	117±19.8
Moderate	19	21.3		
High	70	78.7		

**Table 2:** Frequency distribution of healthcare leadership effectiveness dimensions among managers and their deputies in primary healthcare centers in Madinah/KSA (n=89) Note: The cutoff point was calculated using the demarcation threshold formula: {(total highest score-total lowest score)/2} + total lowest score) [15].

Variables	Frequency	Percent	Min-Max	Mean±SD
Vision creation				
Low	19	21.3	9-25	20.5±3.8
High	70	78.7		
Develop followership				
Low	23	25.8	11-25	20.9±3.6
High	66	74.2		
Implement vision				
Low	15	16.9	10-25	21.7±4.7
High	74	83.1		
Total leadership effectiveness				
Low	18	20.2	33-75	62.7±10.2
High	71	79.8		

**Table 3:** Factors associated with healthcare leadership effectiveness among managers and their deputies in primary healthcare centers in Madinah/KSA (n=89) *Significant.

Variables	Low healthcare leadership effectiveness, N=18	High healthcare leadership effectiveness, N=71	p-Value
Age groups in years			
30-34	2 (11.1%)	3 (4.2%)	0.7
35-40	7 (38.9%)	26 (36.6%)	
41-45	4 (22.2%)	28 (39.4%)	
>45	5 (27.8%)	14 (19.7%)	
Gender			
Male	18 (100%)	66 (93%)	0.3
Female	0	5 (7%)	
Marital status			
Single	0	1 (100%)	0.7
Married	18 (20.9%)	68 (79.1%)	
Divorced	0	2 (100%)	
Education			
Diploma	13 (72.2%)	37 (52.1%)	0.4
Secondary education	1 (5.6%)	4 (5.6%)	
Bachelor's	2 (11.1%)	23 (32.4%)	
Postgraduate	2 (11.1%)	7 (9.9%)	
Family size			
1-4	6 (33.3%)	21 (29.6%)	0.8
5-7	8 (44.4%)	37 (52.1%)	
>7	4 (22.2%)	13 (18.3%)	
Current work position			
PHC manager	8 (44.4%)	44 (62%)	0.3
PHC manager deputy	10 (55.6%)	27 (38%)	
Work experience in years			
< 5 years	1 (5.6%)	1 (1.4%)	0.06
5-15 years	10 (55.6%)	22 (31%)	
>15 years	7 (38.9%)	48 (67.6%)	
Monthly salary in Saudi riyal			
5000 to 9999 SAR	3 (16.7%)	9 (12.7%)	0.9
10,000 to 14,999 SAR	7 (38.9%)	29 (40.8%)	
15,000 to19,999 SAR	6 (33.3%)	23 (32.4%)	
>20,000	2 (11.1%)	10 (14.1%)	
Leadership style			
Democratic	16 (88.9%)	63 (88.9%)	0.7
Autocratic	0	2 (2.6%)	
Laissez-faire	2 (11.1%)	6 (8.5%)	
Courses in leadership and management			
Yes	10 (55.6%)	54 (76.1%)	0.2
No	8 (44.4%)	17 (23.9%)	
Emotional Intelligence			
Low	0	0	0.001*
Moderate	13 (72.2%)	6 (8.5%)	
High	5 (27.8%)	65 (91.5%)	

Furthermore, while all leadership styles correlated with high emotional intelligence scores and leadership effectiveness, the differences were not statistically significant. Table 4 shows that a strong positive correlation was found between healthcare leadership effectiveness and emotional intelligence (r=0.75; p=0.001) but no significant relationship was observed with age or work experience. Binary logistic regression analysis indicated that participants with high emotional intelligence were 1.13 times more likely to demonstrate effective healthcare leadership (OR=1.13; 95% CI: 1.068-1.197) than their lower emotional intelligence counterparts were.

**Table 4:** Correlation between healthcare effective leadership score, age, work experience in years, and emotional intelligence score *Significant.

Variables	Healthcare effective leadership	
	r	p-Value
Age	-0.003	0.9
Work experience in years	0.12	0.3
Emotional intelligence	0.75	0.001*

## Discussion

This study's findings underscore the importance of strong leadership in the healthcare sector, particularly in PHC centers in Madinah, Saudi Arabia. Consistent with global healthcare trends, our study reveals a high level of healthcare leadership effectiveness among PHC managers and their deputies, with an emphasis on vision creation, implementation, and developing followership. These results align closely with those of a Sri Lankan study [18], which reported similarly high levels of leadership effectiveness, and contrast with studies in Addis Ababa and the North Shoa zone, which demonstrated lower levels of leadership effectiveness [14,19]. The greater effectiveness of our study may be attributed to the significant reforms in Saudi Arabia's PHC system, particularly under the PHC reform roadmap (2016-2020) and Vision 2030, which prioritized strengthening PHC governance and leadership through targeted training for PHC managers [11].

Our study also shows that leadership styles vary, and most participants favored democratic leadership. This finding is consistent with the research indicating that democratic leadership is correlated with greater effectiveness in healthcare settings [8]. Contrary to studies in the Kafaa zone and by Teame et al. [14,20], which showed negative impacts of autocratic and laissez-faire leadership on organizational performance, our study did not indicate significant associations between leadership styles and healthcare effectiveness. This discrepancy could be due to the majority of our study's participants receiving leadership and management training, which enabled them to implement more effective leadership practices.

Additionally, the majority of our study participants were middle-aged males with substantial work experience; however, these factors did not significantly influence leadership effectiveness. This observation contrasts with the findings of an Ethiopian study [14], which revealed a positive correlation between experience and effective leadership, and a Nigerian study, which reported mixed findings regarding the impact of experience on leadership skills [21].

A key finding of our study is the strong positive correlation between emotional intelligence and healthcare leadership effectiveness, corroborating similar findings from studies in Egypt and Sri Lanka [18,22]. This finding underscores the crucial role of emotional intelligence in effective healthcare leadership. Interestingly, while most participants exhibited high emotional intelligence, they lacked self-awareness, indicating potential improvements in leadership training programs.

The limitations of this study include potential response bias such as demand bias and cross-sectional design, and suggest the need for further research to validate these findings and explore additional factors influencing healthcare leadership effectiveness.

## Conclusions

In conclusion, our results suggest that effective healthcare leadership in PHC centers in Madinah is influenced predominantly by emotional intelligence and leadership training rather than by demographic factors such as age or years of experience. These insights could inform future interventions and policy developments aimed at enhancing leadership capabilities in the healthcare sector.
